# Ethics of Early Clinical Trials of Bio-Artificial Organs

**DOI:** 10.3389/ti.2022.10621

**Published:** 2022-07-06

**Authors:** Eline M. Bunnik, Dide de Jongh, Emma Massey

**Affiliations:** Erasmus MC, University Medical Centre Rotterdam, Rotterdam, Netherlands

**Keywords:** regenerative medicine, tissue engineering, research ethics, first-in-human clinical trials, bio-artificial organs, ethics

## Abstract

Regenerative medicine is the new frontier in the field of organ transplantation. Research groups around the world are using regenerative medicine technologies to develop bio-artificial organs for transplantation into human patients. While most of this research is still at the preclinical stage, bio-artificial organ technologies are gearing up for first-in-human clinical trials in the not-too-distant future. What are the ethical conditions under which early-phase clinical research of bio-artificial organs can be conducted safely and responsibly? What lessons can be learned from prior experiences with early-phase clinical trials in adjacent fields of research? This is a Meeting Report of an online international workshop organised in the context of the Horizon 2020-funded VANGUARD project, which is developing a bio-artificial pancreas for the treatment of patients with type 1 diabetes.

## Introduction

Although the advancement of medicine calls for clinical research on innovative medical treatments and technologies, early-phase clinical trials are known to be risky and ethically challenging. First-in-human trials especially are associated with serious—predictable and unpredictable—risks for research participants. To justify exposure of volunteers to the risks and burdens of participation in early-phase clinical trials, the research and the resulting intervention must have clear scientific and societal value (1). Today, research groups around the world are developing new applications of regenerative medicine in pre-clinical research settings for the purposes of organ transplantation. Tissue engineering, 3D bio-printing, and organoid technologies are used to generate bio-artificial organs for transplantation into human recipients (2). These technologies might save or improve patients’ lives, and become part of a solution to the problem of donor organ shortage. In the not-too-distant future, they are expected to be ready to be tested in human research participants. It will be challenging for researchers and research ethics committees (RECs) to determine when, and under what conditions, these applications will be ready to make the leap to early-phase clinical research, in a safe and responsible manner.

On 3rd February 2022 an online meeting was held to bring together ethicists, researchers, and clinicians to discuss the ethics of early-phase clinical trials in regenerative medicine in transplantation. The meeting was organised in the context of the VANGUARD project, a European research project which aims to generate a vascularized and immune-protected bio-artificial pancreas that can be transplanted into non-immunosuppressed type 1 diabetes patients.^1^ This project is one of 14 projects funded by the European Commission Horizon 2020 programme “Regenerative medicine: from new insights to new applications”.^2^ Representatives of other recipients of grants from this call were invited to attend the meeting. The meeting was announced on the website and in newsletters of the European Society for Organ Transplantation (ESOT), and open to the public. In total, 102 people registered, and 74 people attended the meeting. The meeting commenced with a keynote lecture by Jonathan Kimmelman, Professor of Biomedical Ethics and Social Sciences of Medicine at McGill University in Montreal, Canada, and was followed by three brief presentations on three bio-artificial organ technologies, and a panel discussion.

## A Theory of—Ethically Responsible—Clinical Translation

Kimmelman laid out his theory of clinical translation in a lecture titled “How to think about the ethics (and the science) of first-in-human trials”. Phase I clinical trials, he said, are among the most vexing challenges in medical research ethics. He brought to mind some of the numerous cases in which either fully or relatively healthy volunteers had died from participating in first-in-human clinical trials, including gene therapy trials in the late 1990s (3). Yet for the advancement of medicine, such trials must be launched.

Kimmelman’s theory is as follows: all drugs, surgeries, vaccines, and devices are “born guilty”; they are poisons, toxins. In one of his papers, Kimmelman cites Paracelsus: “All things are poison, and nothing is without poison; only the dose permits something not to be poisonous” (4). Only by learning to understand how these poisons can be used to target medical conditions in patients, safely and effectively, they can be transformed into technologies of clinical utility. It is by going through the process of clinical translation, that poisons are converted into putative therapeutic interventions.

The process of clinical translation takes time and effort. It consists of two steps. First, we must identify the configuration of materials, practices and beliefs—which Kimmelman calls the “intervention ensemble” (5)—that we must combine with a pharmacological agent or another type of medical technology, to unlock its clinical utility. This includes finding the optimal dosage, mode of delivery, timing and frequency of administration, but also, for instance, knowing what accompanying therapeutic regimen to administer (e.g., immune-suppression), what side effects to look out for, which patients with which comorbidities to exclude, or, in the case of bio-artificial organs, what materials to use, how to assemble or combine them, and who, how, where and how much of them to transplant. Early-phase clinical trials are focused mainly on building this intervention ensemble, on exploring and establishing the approximately optimal conditions in which the investigational treatment will have the desired effects without having the undesired side effects. Second, in later-phase clinical trials, the intervention ensemble must be evaluated rigorously, ideally within randomised controlled trials, in order to demonstrate sufficient efficacy and safety and obtain marketing approval by regulatory authorities. In our online meeting, the focus was on early-phase clinical research.

Kimmelman suggested that there are several moral premises that ought to underwrite clinical translation. First, those of us who are involved in clinical research should maximise “moral efficiency,” that is, for every medical breakthrough, we should minimise welfare loss. Thus, we should minimise the number of patients that are exposed to the risks and harms of research participation. Second, we must ensure that we generate information that healthcare systems need to support the practice of efficient and cost-effective medicine. We need to know how to use and how not to use medical technologies. This also means that we must understand the relative or incremental value of a new technology as compared to other, existing therapeutic approaches. Third, we should acknowledge that clinical translation is not like a pipeline but more like a web (6), a dense network of collaboration among various stakeholders who must trust one another. For example, research participants should be able to trust researchers when “lending their bodies to research.” Therefore, we must advance rules and practices that protect and maintain the stability of these networks. Kimmelman believes that we may not currently be meeting these moral requirements in full. He discussed three areas of concern: risk-benefit assessment, subject selection, and informed consent.

### Risk-Benefit Assessment

When sponsors and researchers are considering to set up a clinical trial, and when research ethics committees (RECs) are evaluating a protocol for a clinical trial, an assessment must be made of the balance between risks and potential benefits associated with the trial. There are widespread but mistaken assumptions about risk-benefit assessment, according to Kimmelman. For instance, while it is generally assumed that sponsors would not initiate trials unless there were a good prospect of success, in reality, they may do so in the absence of such prospect. Also, it is assumed that regulatory authorities will make risk-benefit assessments before trials are launched. In practice, however, regulatory authorities defer to RECs for assuring that the “risks to subjects are reasonable in relation to anticipated benefits, if any, to subjects, and the importance of the knowledge that may be expected to result” from trials (7). Moreover, there is little guidance on how RECs should judge whether an intervention is promising enough to launch a clinical trial (8–10). At minimum, RECs require solid pre-clinical evidence, for instance, evidence that is confirmed in multiple relevant animal models. The International Society for Stem Cell Research (ISSCR) lists key design principles for pre-clinical studies for the generation of rigorous evidence to support decision-making about clinical trials (11).

### Patient Selection

In selecting subjects for participating in early-phase clinical research, a balance must be sought between the dual aims of maximising moral efficiency and generating useful evidence. Patients with late-stage or refractory disease will have less to lose—but possibly also less to gain—than healthy volunteers or patients with more recent disease onset. Starting with patients who are more severely ill—and without satisfactory alternatives—helps to avoid dramatic outcomes, and therewith, crises of confidence, such as the crisis of confidence in the gene therapy field in the late 1990s (12). When designing early-phase clinical trials, researchers should consider the effects of patient selection on the maintaining of trust and the stability of the collaborative networks needed for clinical translation.

### Informed Consent

Most patients are taking part in early-phase clinical trials in the hope of gaining medical benefit, even though, as part of the informed consent process, they are informed that it is uncertain whether they will benefit. Kimmelman suggested that researchers should be more forthright to research participants, and explain to them that major benefits are “highly unlikely” (13), and that patients are “more likely to experience side effects than benefit medically.” Patients should understand that by participating in early-phase clinical research, they contribute to welfare gain to society but are likely to experience welfare loss themselves.

## Bioartificial Organ Technologies: Three Examples

Following the lecture, three examples were presented by junior researchers Ollala Iglesias García, Ary Marsee, and Dide de Jongh of bio-artificial organ technologies that are currently under pre-clinical development within the context of the aforementioned Horizon2020-call.

In the project BRAVE, led by researchers of the University of Navarra, regenerative medicine and 3D-bioprinting are combined with computational modelling to develop a biological ventricularassist device, which is meant to provide lifelong support to patients with ischemic heart disease. The researchers are aiming to bring the device “as close to the bedside in the shortest time possible”.^3^ The device consists of a 3D-printed microfibre scaffold seeded with human induced pluripotent stem cells, to be integrated in the patient’s heart and restore cardiac function. Computational modelling is used to assess cardiac geometry and tissue mechanics, such that the design of the assist device can be tailored to the individual patient’s heart.

Researchers of the project OrganTrans, which is coordinated by the Swiss Centre for Electronics and Microtechnology (CSEM), are building a platform for liver tissue engineering as a “disruptive alternative to donor organs” for treating patients with chronic end-stage liver diseases.^4^ The platform uses stem cells that are derived from the patient’s residual healthy liver tissue, which self-assemble and self-organize into liver organoids. Organoids are supported by 3D bio-printed scaffolding made up of synthetic hydrogel and vascular networks made using endothelial cell ink, to reconstruct functional liver tissue for transplantation into patients.

Finally, in VANGUARD, which is led by researchers at the University of Geneva, a bio-artificial pancreas is being developed for the treatment of type 1 diabetes.^5^ The bioartificial organ is composed of islets of Langerhans from deceased donors, an extra-cellular matrix consisting of genome-edited human amniotic endothelial cells derived from donated placentas to protect islet cells against inflammatory and hypoxic damage and to accelerate engraftment, and patient-own blood outgrowth endothelial cells for vascularisation and immune-protection.

## Panel Discussion

In the panel discussion, Kimmelman was joined by three senior representatives of the above projects, Manuel María Mazo Vega (BRAVE), Mariana Pacheco Blanco (OrganTrans) and Ekaterine Berishvili-Berney (VANGUARD), and Anne-Floor de Kanter of Utrecht University, who is pursuing a PhD in ethics of regenerative medicine. During the panel discussion, several ethical issues were raised in response to the presentations of the three new bio-artificial organ technologies currently under development. Four issues that are most relevant to early-phase clinical research on bio-artificial organs are briefly discussed below.

### Are Bioartificial Organs Special?

The ethical issues arising in early-phase clinical research on bio-artificial organ technologies, it was generally agreed by the panel, are not entirely novel or unique. Lessons can be learned from prior experiences in other areas in medicine, including other applications of regenerative medicine and gene therapy (14). However, there are not only similarities, but also differences, between the transplantation of bioartificial organs and, for instance, the administration of pharmaceutical agents or cell and gene therapies, or the implantation of (non-biological) medical devices. Firstly, transplantation of bio-artificial organs requires surgery, that is, making skin incisions, entering the body, and making changes to the anatomy of the patient. Thus, it is invasive—more so than pharmaceutical agents, which may be taken orally, or cell and gene therapy, which may be injected or infused. Secondly, as the “product” is composed of biological materials, and “metabolically active” (15), it may integrate with the body of the recipient and develop within the body over time (16). Consequently, the treatment is likely to be irreversible (15)—more so than treatment using non-biological medical devices, which can be removed integrally. Thirdly, bio-artificial organs are complex: they may be composed of biological materials derived from various sources. To develop the bio-artificial pancreas, for instance, researchers need access to biological materials derived from deceased donors and new mothers—raising ethical issues known from the field of organ transplantation generally, including informed consent from donors and the crucial importance of maintaining—and deserving—public trust. Bio-artificial organs are complex also in the sense that—in contrast to cell and gene therapies—they are organised in three-dimensional space. There is little experience yet with exploring the intervention ensemble in terms of requirements for the three-dimensional organisation of tissues.

Finally, what makes the coupling of regenerative medicine with transplant medicine potentially revolutionary is its aspect of “personalisation”: by using patients’ own cells to generate organs for transplantation, the major hurdle of the need for patients to take lifelong immune-suppressive medications and the associated long-term complications, can be overcome (2). As each bio-artificial organ is personalised, however, each “product” is different. It cannot be “constructed in uniform batches according to well defined standards to the same extent as medical devices or medicinal products” (16). This renders the generation of evidence of the product’s safety and efficacy more difficult. Personalised technologies may need to be evaluated—and regulated—not as medicines, but as health services (17). In a services-based regulatory model, it would not be the product, but rather the service that is evaluated and approved for use. In OrganTrans, for instance, it would not be the personalised liver organoids that are approved for use, but the platform for liver tissue engineering—not the bio-artificial organ itself, but the methods used for its creation.

It will be clear to the reader that none of these characteristics—invasiveness, integration and irreversibility, complexity, and personalisation—are unique to bio-artificial organs. In fact, most Advanced Therapy Medicinal Products (ATMPs), a category of “medicines” for the European Medicines Agency (EMA) that includes gene therapy, somatic-cell therapy, and tissue-engineered medicines, will have one or more of these characteristics.^6^ What is new about bio-artificial organs for transplantation, is that these characteristics are combined in one technology, and that they accumulate and may interact, thus heightening the ethical sensitivity of their application.

### Social Value

In the panel discussion, there was a strong focus on the social value of research and development of bio-artificial organ technologies. This is not surprising, as three of the panellists were involved in research projects funded by a Horizon 2020 programme that is meant to stimulate clinical translation of regenerative medicine technologies.^7^ Panellists were intent not only on advancing science, but first and foremost on developing technologies that may benefit patients. Bio-artificial technologies should lead to improvements in health or well-being of future patients.

Whether new technologies succeed in contributing to health or well-being depends not only on the safety and efficacy of the technology itself, but also on the scientific and societal context in which the technology is being developed. The social value of a new technology is defined as its clinical benefit to future patients relative to alternatives that may already be approved for marketing (18), or that are being developed in parallel, and may become available in the near future. For instance, in recent years, due to significant advances in continuous glucose monitoring and continuous subcutaneous insulin infusion technologies, treatment of type 1 diabetes has become more effective—also in patients at higher risk of hypoglycaemia or with hypoglycaemia unawareness—and less burdensome (19). Also, there are new classes of immunotherapeutic medicines, such as anti-CD3 antibodies, which seems to delay progression to type 1 diabetes in high-risk research participants (20), and possibly to halt the disease in newly diagnosed patients, although the evidence is not convincing (21). While these technological advances will have improved the treatment of many type 1 diabetes patients, there may be subgroups of patients who cannot be adequately treated using pharmaceuticals or automated sensor-pump combinations, and who thus qualify for more invasive interventions, ranging from conventional islet or pancreas transplantation to, possibly, bio-artificial organ technologies. Adding social value thus entails identifying the groups of patients who will benefit the most.

Further, social value can only be realized when new technologies actually reach those groups of patients who will benefit the most. Bio-artificial organs might help to reduce the global disease burden associated with ischemic heart disease, end-stage liver disease, or type 1 diabetes, but only if they can be accessed by patients who need them, including patients in developing countries. Tissue engineering and 3D-bioprinting technologies, however, require highly specialised personnel, equipment, information technology, and laboratory facilities, which may not in place everywhere in the world. Researchers and developers in developed countries should think about how bio-artificial organs—or rather the technologies used to generate them—can be distributed to other geographical areas.

Accessibility implies not only availability, but also affordability. The prices of bio-artificial organs are likely to be high (22). This may not be due to material costs: the bio-artificial pancreas, for instance, is composed, among other things, of patient-derived material, which should be free, and placenta, which is medical waste, and can be procured at low cost. However, clinical development of bio-artificial organ technologies will require major financial investments that are beyond the reach of research groups themselves (23). Manufacturers need to recover the costs of development and reward their investors within the—often limited—timeframe of market exclusivity (24), thus driving up the prices of new medical technologies. Over time, however, as patents expire and monopolies are rescinded, prices may decrease.

### Randomised Controlled Trials

While the focus of our meeting was on early-phase clinical research, it was felt that researchers should already be anticipating ethical issues that will arise in later-stage clinical research, in which rigorous evidence must be generated of the clinical utility of bio-artificial organ technologies. To optimize the scientific validity of later-stage research, researchers should ideally conduct randomised controlled trials.

However, in the field of surgery, innovation has traditionally occurred mainly through gradual improvements on modi operandi in operating theatres, and it has not been customary for surgeons to conduct randomised controlled trials (25). Also, there is much less of a paradigm for funding clinical trials in surgery than there is for funding drug trials. Moreover, the design requirements for late-stage trials of bio-artificial organs are not clear, notably, in relation to the choice of a comparator. To ensure double blinding, the comparator should ideally be sham (or placebo) surgery. Patients are known to respond strongly to placebo in clinical trials of (minimally invasive) surgery (26). Sham surgery, however, inherently implies physical harm and risks (27), and is not commonly used (28). It will likely be ethically acceptable to expose research participants to sham surgery only in the context of clinical equipoise (29), when the harms and risks of sham surgery may be deemed justifiable (27). This will probably not be the case in transplantation trials of vital bio-artificial organs, such as livers or hearts, as withholding standard of care may lead to severe illness or even death.

Panellists indicated that RECs had an important role to play in assessing the risks and benefits of clinical trials of bio-artificial organs, helping guide selection of research participants (30), evaluating and improving upon study design, and overseeing the adequacy of informed consent models. Panellists expressed the concern that RECs are currently not fully equipped for their role in evaluating protocols for clinical trials of regenerative medicine applications in organ transplantation, and that RECs must be strengthened, for instance through attracting and including expertise in regenerative medicine and organ transplantation.

### Public Dialogue

Finally, panellists believed that researchers should communicate carefully about bio-artificial organs with patients and lay audience, without fuelling hype or crushing hope. Over the years, scientific advances in regenerative medicine have been surrounded by much hype and great expectations (31). After news about regenerative medicine technologies is reported in the media, panellists report, patients tend to ask their clinicians if and when the new treatment will be available to them, even though it may still take years—or even decades—for the treatment to be implemented in the clinic. It is important for researchers to stress that bio-artificial organ technologies are still being investigated in pre-clinical research settings, and to do so in language that is comprehensible to the public. Public dialogue is seen as serving two aims: firstly, to build and maintain (or even restore) trust in bio-artificial organ technology. This is necessary, as earlier research on bio-artificial organs in transplantation has raised some negative attention and scientific and clinical controversy (24). Secondly, clinical research can only be conducted as long as patients are willing to participate in research and there is general support within societies for the scientific endeavour. Participants in trials of bio-artificial organs may need to be followed up over long periods of time to monitor long-term adverse effects or complications, which requires long-term commitment. Researchers must therefore enter into long-term trust relationships with research participants. Representatives of the three European projects report that they have included patient organizations in their advisory boards, to ensure that patient voices are heard and used to help guide research questions, research design, and knowledge utilisation.

## Concluding Remarks

Early-phase clinical transplantation trials of bio-artificial organs raise ethical issues in relation to risk-benefit assessment, patient selection, and informed consent. Although these issues are not new, clinical translation of bio-artificial organ technologies does present a new constellation of ethical challenges not found in other areas of clinical research. There are several ethical challenges that must either be thought through or acted upon. Researchers should think carefully about trial design, patient selection, and informed consent. To ensure that patients provide truly informed consent for early-phase clinical trials, the potential benefits of research participation should not be overstated. Transparent communication about risks and benefits helps to restore and maintain the trust of patients and publics alike. Clinical translation of rapidly advancing regenerative medicine technologies to the field of organ transplantation may be challenging, high-risk, laborious, and of uncertain commercial value, but without the effort, patients in need of organ replacement therapy will not be able to reap the fruits of these advancements. Researchers and manufacturers may need to think about ways of making their products or their technologies accessible to patient populations around the world, which might require the involvement of multi-stakeholder networks. Researchers must engage patient communities and the general public in clinical research to ensure that new bio-artificial organ technologies are aligned with patients’ needs and preferences, and that societal concerns are adequately addressed. Finally, research ethics committees must be strengthened by including specific expertise in regenerative medicine and organ transplantation, so that they can help ensure that early-phase clinical trials of bio-artificial organs are conducted in a safe and ethically responsible manner.

## VANGUARD Consortium Partners

Ekaterine Berishvili, Laura Mar Fonseca, Fanny Lebreton, Kevin Bellofatto, Juliette Bignard, University of Geneva, Department of Surgery, Geneva, Switzerland. Jochen Seissler, Leila Wolf-van Buerck, Mohsen Honarpisheh, Yichen Zhang, Yutian Lei, Monika Pehl, Diabetes Centre, Ludwig-Maximilians University Munich, Germany. Antonia Follenzi, Chiara Borsotti, Simone Merlin, Department of Health Sciences, University of Piemonte Orientale, Novara, Italy. Lorenzo Piemonti, Antonio Citro, Silvia Pellegrini, IRCCS Ospedale San Raffaele, Diabetes Research Institute, Milano, Italy. Olivier Thaunat, Lyon Claude Bernard University, Dept. Transplantation, Nephrology and Clinical Immunology, Lyon, France. Antonia J. Cronin, King’s College, London, United Kingdom. Devi Mey, Chiara Parisotto, Giovanna Rossi, European Society for Organ Transplantation, Padova, Italy. Patrick Kugelmeier, Petra Wolint, Markus Mühlemann, Karolina Pal-Kutas, Kugelmeiers AG, Erlenbach, Switzerland. Marco Cavallaro, Julia Götz, Jeanette Müller, Accelopment Switzerland Ltd.

## Footnotes

1 VANGUARD. New generation cell therapy: bioartificial pancreas to cure type 1 diabetes. https://vanguard-project.eu/(Accessed 4 May 2022).

2 European Commission. Regenerative medicine: from new insights to new applications. https://cordis.europa.eu/programme/id/H2020_SC1-BHC-07-2019 (Accessed 4 May 2022).

3 BRAVE. A therapy for life to restore the patient’s heart function. https://projectbrave.eu/(Accessed 4 May 2022).

4 OrganTrans. Controlled organoids transplantation as enabler for regenerative medicine translation. https://organtrans.eu/ (Accessed 4 May 2022).

5 VANGUARD. New generation cell therapy: bioartificial pancreas to cure type 1 diabetes. https://vanguard-project.eu/ (Accessed 4 May 2022).

6 European Medicines Agency (EMA). Advanced therapy medicinal products: overview. https://www.ema.europa.eu/en/human-regulatory/overview/advanced-therapy-medicinal-products-overview (Accessed 4 May 2022).

7 European Commission. Regenerative medicine: from new insights to new applications. https://cordis.europa.eu/programme/id/H2020_SC1-BHC-07-2019 (Accessed 4 May 2022).
